# Classic Signaling Pathways in Alveolar Injury and Repair Involved in Sepsis-Induced ALI/ARDS: New Research Progress and Prospect

**DOI:** 10.1155/2022/6362344

**Published:** 2022-06-18

**Authors:** Wenli Li, Duo Li, Yuansen Chen, Halidan Abudou, Haiwang Wang, Jinxia Cai, Yiping Wang, Ziquan Liu, Yanqing Liu, Haojun Fan

**Affiliations:** ^1^Institute of Disaster and Emergency Medicine, Tianjin University, Tianjin, China; ^2^Wenzhou Safety (Emergency) Institute, Tianjin University, Wenzhou, China

## Abstract

Sepsis is a common critical clinical disease with high mortality that can cause approximately 10 million deaths worldwide each year. Acute lung injury (ALI) or acute respiratory distress syndrome (ARDS) is a common clinical complication of sepsis, which occurs primarily as diffuse alveolar injury, hypoxemia, and respiratory distress. The mortality rate of ALI/ARDS is as high as 30%-40%, which greatly endangers human health. Due to the unclear pathogenesis of ALI/ARDS, its treatment is still a worldwide problem. At present, clinical treatment mainly relies on lung-protective ventilation, prone position ventilation, and fluid management. However, there is a lack of effective and specific treatment measures. In recent years, domestic and foreign scholars have committed to basic research on ALI/ARDS, trying to further clarify its pathogenesis and find new targets and methods for the treatment of ALI/ARDS. In this review, we summarize the signaling pathways related to alveolar injury and repair in sepsis-induced ALI/ARDS and their latest research progress. They include the NF-*κ*B, JAK2/STAT3, mitogen-activated protein kinase (MAPK), mTOR, and Notch signaling pathways. Understanding the molecular mechanisms of these signaling pathways in sepsis-induced ALI/ARDS may provide new targets and ideas for the clinical treatment of this disease.

## 1. Introduction

**Figure 1:**
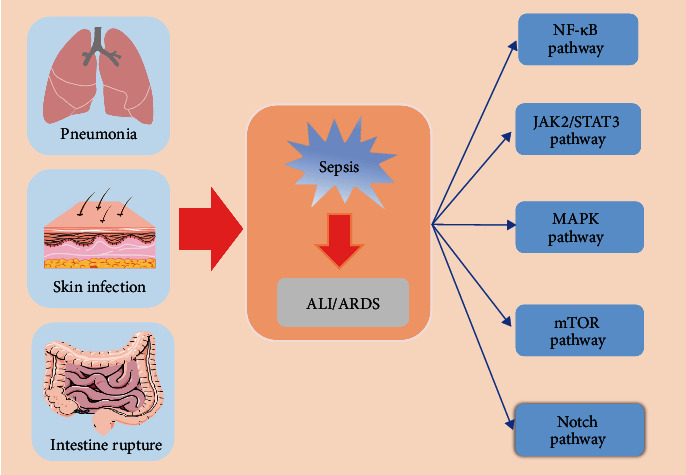
Overview of sepsis-induced ALI/ARDS. Pneumonia, local infections of the skin, and intestinal commensal bacteria leak into the blood may lead to the development of sepsis and ALI/ARDS. Multiple signaling pathways are activated during the progression of sepsis to ALI/ARDS.

Sepsis is a fatal organ dysfunction caused by immune dysfunction following infection of the host [1, 2]. It is a serious illness commonly encountered in clinical practice. Annually, there are approximately 48.9 million sepsis cases and 11 million sepsis-related deaths worldwide [3]. Cytokine storm during sepsis can lead to unresolvable lung inflammation and the development of acute lung injury (ALI) or acute respiratory distress syndrome (ARDS). It causes irreversible damage to the lung and high mortality. Sepsis-induced ALI/ARDS can occur as a consequence of both direct lung injury caused by lung epithelial injury and indirect lung injury caused by endothelial cell injury [4–7]. Compared with ALI/ARDS caused by other causes, sepsis-related ALI/ARDS has a higher mortality rate of 30% to 40% [7–10]. In the progression of sepsis to ALI/ARDS, cytokines can mediate the aggregation and infiltration of a large number of immune cells into lung tissue to activate intracellular signal transduction pathways and release a large number of cytokines. Inflammatory cells are constantly activated, forming a vicious cycle and ultimately resulting in a cytokine storm [11–13]. Consequently, cytokine storm is a significant factor in the progression of sepsis to ALI/ARDS. A variety of inflammatory cells are activated in this process and release a large number of inflammatory mediators. They destroy the integrity of the alveolar-capillary endothelial barrier structure, with causing neutrophil infiltration and diffuse pulmonary edema. During the process, they activate multiple intracellular signaling pathways (Figure 1). However, the exact pathogenesis of the disease is still unclear. And its treatment remains a global problem. Moreover, there are no specific drugs available for the treatment of ALI/ARDS. In this review, we summarize the roles and mechanisms of five signaling pathways in regulating alveolar injury and repair in sepsis-induced ALI/ARDS.

## 2. Signaling Pathways Related to ALI/ARDS

### 2.1. NF-*κ*B Pathway

**Figure 2:**
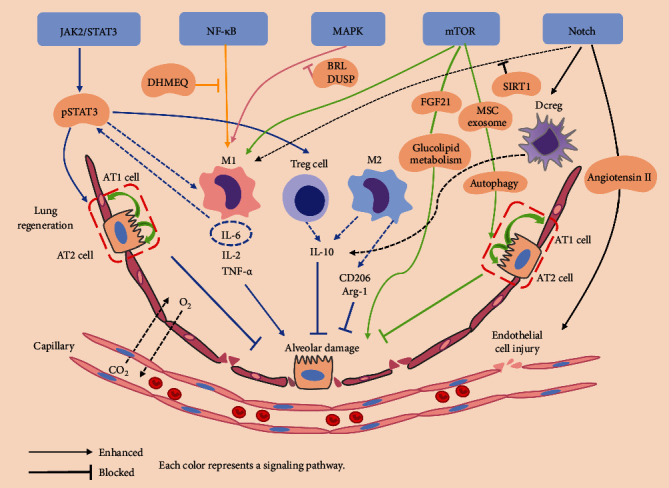
Regulation of five signaling pathways on alveolar injury and repair during sepsis-induced ALI/ARDS. The activation of STAT3 can enhance the effect of proinflammatory macrophages (M1) on cytokine storm. The activation of STAT3 can also activate Treg cells to increase the secretion of IL-10 to promote the proliferation and differentiation of AT2 cells (lung regeneration) during the alveolar injury and repair. NF-*κ*B pathway recruits M1 to produce cytokine (TNF-*α*, IL-6), which promote the cytokine storm and the development of ALI/ARDS. Inhibition of MAPK signaling pathway by dual-specificity phosphatase (DUSP) can inhibit inflammatory response in ALI/ARDS. The mTOR signaling pathway and Notch signaling pathway can promote M1 to produce cytokine to aggravate the inflammation of ALI/ARDS. FGF21 increases the incidence of ALI/ARDS by regulating glucose and lipid metabolism through the mTOR signaling pathway. MSC exosomes can inhibit ALI/ARDS by inducing autophagy and pulmonary regeneration. The Notch signaling pathway can also promote Dcreg cells to produce IL-10 to inhibit ALI/ARDS. DHMEQ: dehydroxymethylepoxyquinomicin; M1: proinflammatory macrophages; M2: anti-inflammatory macrophages; DUSP: dual-specificity phosphatase; BRL: BRL-44408 maleate; FGF21: fibroblast growth factor 21; Arg-1: Arginase-1; Treg cell: regulatory T cell; SIRT1: Sirtuins1; DCregs: regulatory dendritic cells.

NF-*κ*B is a dimeric transcription factor in B lymphocytes. It is named for its specific binding to the B-ring enhancer of the *κ* light immunoglobulin chain. As a family of transcription factor proteins, NF-*κ*B is not transcriptionally active when it binds to the NF-*κ*B inhibitor (IkB) in the cytoplasm in a resting state. When externally stimulated, IkB kinase (IKK) is activated to detach and degrade the IkB protein from NF-*κ*B. Then, NF-*κ*B binds to specific target sites of DNA in the nucleus to initiate the transcription and expression of target genes, releasing interleukin 2 (IL-2), interleukin 6 (IL-6), and other inflammatory factors [14]. NF-*κ*B pathways include classical and nonclassical pathways [15]. The common activated form of the conventional signaling channel is the dimer P65/P50. NF-*κ*B is implicated in processes such as the inflammatory response, the host immune response, cell adhesion, growth signaling, cell proliferation, cell differentiation, and apoptotic defense. Studies have shown that the NF-*κ*B pathway plays significant roles in a variety of inflammatory respiratory diseases, including pulmonary fibrosis, asthma, lung cancer, and obstructive pulmonary disease [16]. When the body is stimulated externally, NF-*κ*B induces the expression of several proinflammatory cytokine genes. These proinflammatory cytokines further activate the NF-*κ*B signaling cascade in an autocrine manner to amplify the inflammatory response [17]. For sepsis-induced ALI/ARDS, new drug therapy studies related to this pathway have shown some success. For example, butorphanol significantly reduced sepsis-induced lung tissue injury and mortality by promoting M2 macrophage polarization and inhibiting M1 macrophage polarization through the NF-*κ*B pathway in a mouse model of sepsis-induced ALI [18]. M1 macrophages produce proinflammatory factors, such as reactive oxygen species and reactive nitrogen intermediates, as well as the cytokines tumor necrosis factor-alpha (TNF-*α*) and IL-6 [19, 20]. M2 macrophages are polarized and produce anti-inflammatory cytokines and other substances, such as interleukin-10 (IL-10), Mrc1 (CD206), and Arginase-1 (Arg-1), which are involved in wound healing and tissue repair [20, 21]. Aspirin has been shown to treat sepsis or ARDS by inhibiting NF-*κ*B mobilization and monocytic adherence in stimulated human endothelial cells [22, 23]. Furthermore, preactivated and catabolic-deformed platelets prevent ARDS from complicating sepsis by inhibiting inflammation associated with the NF-*κ*B [24]. Several studies have shown that HIF-1*α* can activate the NF-*κ*B pathway and increase the secretion of cytokines such as TNF-*α* and IL-6 in the early stages of a cytokine storm [25, 26]. In addition, dehydroxymethylepoxyquinomicin (DHMEQ) can inhibit NF-*κ*B activation and reduce inflammatory cytokine expression [27]. As shown in Figure 2, NF-*κ*B pathway can promote autoimmunity and inflammation when it is aberrantly activated in different cell types. Dysregulated NF-*κ*B pathway in endothelial cells can lead to aberrant chemokine production and inflammatory cell recruitment, resulting in excessive inflammation and/or tissue damage. Drugs related to the NF-*κ*B pathway have achieved certain results in patients with the coronavirus disease 2019 (COVID-19), such as proteasome inhibitors (VL-01, bortezomib, carfilzomib, and ixazomib), Bruton tyrosine kinase inhibitors (acalabrutinib), nucleoside analogues (remdesivir), TNF-*α* monoclonal antibodies (infliximab and adalimumab), N-acetylcysteine, and corticosteroids (dexamethasone) [28]. Thus, blocking or inhibiting the NF-*κ*B pathway could be a therapeutic target in a sepsis-induced precursor cytokine storm (Figure 2).

### 2.2. JAK2/STAT3 Pathway

The JAK2/STAT3 pathway is a common pathway for multiple cytokine signaling and is widely involved in multiple biological processes including the inflammatory response and apoptosis [29–31]. Janus kinases (JAKs) include JAK1, JAK2, JAK3, and TYK2. Signal transducers and activators of transcription (STATs) include seven structurally and functionally related proteins: STAT1, STAT2, STAT3, STAT4, STAT5a, STAT5b, and STAT6 [32, 33]. The JAK2/STAT3 pathway has an immunomodulatory role similar to NF-*κ*B pathway in cytokine storms. However, it is inconclusive whether activation of this pathway has a facilitative or inhibitory effect on ALI/ARDS. Several studies have found that the cytokine storm of COVID-19 can be alleviated by inhibiting JAK2/STAT3 pathway, and drugs related to this pathway, such as baricitinib, ruxolitinib, and tofacitinib, have been used in the clinic [34–36]. However, some studies have revealed that the JAK2/STAT3 pathway promotes lung tissue repair function during lung damage [37]. IL-6 has recently received attention due to its important role in the cytokine storm caused by COVID-19 [38]. IL-6 is a central and important marker of the cytokine storm, and its secretion triggers a cascade of amplified inflammatory responses. Several studies have shown that IL-6 activates Janus kinase and phosphorylates STAT3 downstream of it through binding to receptors on the cell membrane via the JAK-STAT pathway, thereby initiating the transcription of STAT3 target genes [39, 40]. It also plays an important role in sepsis-induced ALI/ARDS. Researchers identified STAT3 as a key regulatory gene and an important marker of sepsis-induced ARDS by analyzing whole-blood gene expression profiles of sepsis patients and sepsis-induced ARDS patients during the evolution of their disease [29]. Lignocaine may reduce caecum ligation and puncture- (CLP-) induced lung injury and caspase-11-dependent cellular scorching of ALI mice by reducing STAT3 phosphorylation and regulating the ratio of Tregs and IL-10 expression in lung tissues [41, 42]. In a mouse model of sepsis-induced ALI, the organism may activate the STAT pathway through IL-6 to promote the development of an inflammatory response [43]. In addition, methotrexate (MTX), an anti-inflammatory agent that inhibits the JAK2/STAT3 pathway, has been shown to ameliorate systemic inflammation and lung injury in a rat model of CLP sepsis [44]. Inhibition of STAT3 activity by the small molecule inhibitor LLL12 reduces the infiltration of macrophages and inflammatory cells and protected against lipopolysaccharide- (LPS-) induced ALI [45]. Moreover, JAK2/STAT3 is also involved in the poor prognosis of ARDS patients. It has been shown that IL-6 is involved in muscle dysfunction in ARDS patients via JAK2/STAT3, FOXO3a, and atrogin-1 [46]. The regeneration of the alveolar epithelium is vital for the healing of devastating lung diseases. Recent studies have been conducted to explore the effects of the JAK2/STAT3 signaling pathway on the proliferative capacity of AT2 cells and lung repair function in lung injury. For example, high-throughput and single-cell sequencing of AT2 cells isolated from ALI mice revealed that the STAT3-BDNF-TrkB signaling axis promotes alveolar epithelial regeneration after lung injury [37]. Surface-active C protein is an important surface-active ingredient for the lung, and SPC has been found to inhibit inflammation and promote lung regeneration by reducing JAK2/STAT3 activation during lung repair [47]. Therefore, activating the JAK2-STAT3 pathway has a double effect on the progression of sepsis-induced ALI/ARDS. On the one hand, the overactivation of JAK2/STAT3 induces macrophages to secrete inflammatory factors, making the cytokine storm a cascading amplification effect. On the other hand, the JAK2/STAT3 pathway can promote the proliferation and differentiation of AT2 cells and promote the repair and regeneration of the damaged lung to inhibit the progression of ALI/ARDS (Figure 2).

### 2.3. Mitogen-Activated Protein Kinase (MAPK) Pathway

Mitogen-activated protein kinase (MAPK) is a kind of serine/threonine protein kinases widely found in eukaryotic cells that transduce cells and their nuclei by phosphorylating target proteins through a three-tiered kinase cascade pathway. The pathway is implicated in cellular proliferation, growth, apoptosis, and other processes [48, 49]. Previous studies have shown that the MAPK pathway may have an important pathogenic role in the inflammatory process associated with sepsis-induced ALI/ARDS [50–52]. Studies have shown that a variety of medications inhibit the onset of the LPS-induced inflammatory response by blocking the MAPK pathway or phosphorylation [51, 53, 54]. It has been demonstrated that the oligomeric form of surfactant protein D induces anti-inflammatory effects in M1 subtype macrophages via the calreticulin/p38 MAPK in a mouse model of ALI [55]. Another study showed that ginseng could treat LPS-induced ALI in mice by reducing lung histopathological damage, pulmonary edema, cytokines, and neutrophil aggregation. Using pathway enrichment analysis from the GO and KEGG databases, the phosphatidylinositol 3-kinase-protein kinase B (PI3K-AKT) and mitogen-activated protein kinase (MAPK) pathways were considered critical for ginseng in ALI/ARDS therapy [56]. In addition, downregulation of Toll-like receptor 4 (TLR4) expression and inhibition of extracellular signal-regulated kinase (ERK)1/2 and p38 MAPK activation inhibited the inflammatory responses in LPS-induced ALI [57]. Moreover, immunomodulating nanoparticles have been found to reduce macrophage inflammation by inhibiting the reprogramming of NF-*κ*B and p38 MAPK functions by lactic acid [58]. Blocking the p38 MAPK pathway leads to a shift from proinflammatory apoptosis to noninflammatory apoptosis in macrophages, providing a new guidance for the treatment of ALI/ARDS patients [59]. Inhibiting the JNK pathway may reduce the lung damage from sepsis by reducing apoptosis [60, 61]. In addition, DUSPs dephosphorylate MAPK to control the activity of the MAPK pathway [62]. This suggests that sepsis-induced ALI/ARDS may induce phosphorylation of JNK and p38 MAPK in lung tissue. Specific inhibitors of JNK and/or p38 MAPK may block MAPK signaling, increase serum levels of anti-inflammatory factors, and decrease serum levels of proinflammatory factors, thereby significantly improving lung histopathology and permeability (Figure 2).

### 2.4. mTOR Pathway

Mammalian target of rapamycin (mTOR) is a serine/threonine kinase that regulates many important cellular processes such as cell proliferation, translation, transcription, and autophagy. It has two large physical and functional catalytic subunits. The mTOR complex 1 (mTORC1) plays a key role in cell growth in response to nutrients. And the mTOR complex 2 (mTORC2) controls cell proliferation and survival [63]. AMPK (AMP-activated protein kinase) and TOR (target of rapamycin) pathways are interrelated and antagonistic. When cells lack energy or nutrients, the AMPK pathway is activated and inhibits cell growth. However, when cells have sufficient nutrients, the TOR pathway is activated and promotes cell growth [64]. AMPK acts as an energy receptor in the cell. When intracellular ATP levels are low, this pathway is activated and provides ATP through fatty acid oxidation and autophagy. However, when intracellular ATP levels are high, it can consume ATP by regulating processes such as gluconeogenesis, lipid, and protein synthesis. Fibroblast growth factor 21 (FGF21), as a member of the superfamily FGF, is also a key regulator of glucolipid metabolism [65]. A significant increase in serum of FGF21 levels from baseline is associated with 28-day mortality in intensive care unit (ICU) patients with sepsis and ARDS [66]. Moreover, the AMPK/mTOR pathway is a switch between anabolic and catabolic processes in the cell. Ferroptosis in sepsis-induced acute lung damage has been shown that it can be attenuated by blocking mTOR signaling and autophagy [67]. Clara secretory cell protein (CC16), a natural anti-inflammatory factor in the lung, inhibits lung injury by activating the PI3K/AKT/mTOR/ERK1/2 pathway to promote A549 cell proliferation and inhibit LPS-induced apoptosis [68]. Circulating serum miR-92a was found to be elevated in patients with sepsis-induced ARDS, and miR-92a inhibitors inhibited LPS-induced apoptosis and inflammatory responses via the Akt/mTOR pathway [69]. Furthermore, autophagy is an important intracellular process, and reactive oxygen species (ROS) and reactive nitrogen species (RNS) are the primary intracellular signaling sensors that maintain autophagy [70]. Although the role of autophagy in ALI/ARDS has been controversial, some studies have revealed adverse effects of autophagy on lung injury [71, 72], while other studies have shown protective effects [73, 74]. A recent study showed that mesenchymal stem cell (MSC) exosomes significantly improved LPS-induced ALI by inducing autophagy [75]. In addition, increased oxidative stress downstream of autophagy was found to promote red blood cell distribution width (RDW) by reducing erythrocyte survival and releasing large numbers of premature erythrocytes into the circulation, and RDW has emerged as a new independent prognostic marker in patients with sepsis-induced ARDS [76, 77]. Thus, it is clear that the mTOR signaling pathway is mainly associated with energy metabolism and autophagy is involved in regulating various biological functions in the lung, such as the inflammatory response, DNA damage repair, apoptosis, cell proliferation, and differentiation. Therefore, autophagy plays a crucial role in maintaining the metabolic homeostasis of pulmonary tissues and the development of chronic respiratory diseases. The appearance of pulmonary fibrosis in ALI/ARDS induced by late sepsis may be closely related to this process (Figure 2).

### 2.5. Notch Pathway

Notch pathway is highly conserved from Drosophila to mammals and plays an important role in embryonic development. The receptor and ligand of this signaling pathway are membrane proteins associated with the control of cell proliferation/differentiation and apoptosis through cellular interactions [78]. It is known that the Notch pathway affects various immune and nonimmune cells by regulating cell proliferation and fate. Activation of the Notch signaling pathway induces activated subtype (classically activated macrophage, M1) to promote inflammation, whereas blocking it induces alternative activated macrophage (M2) to suppress inflammation [79–81]. This pathway is essential for the development of various tissues and organs. Notch dysregulation is associated with various lung diseases, especially lung cancer metastases [82–89]. The important regulatory role of Notch signaling in this disease was reported in a recent study in which midkine was found to be positively associated with sepsis-induced lung injury in peripheral blood samples from patients with sepsis and in vitro studies showed that Notch 2 was involved in midkine-induced activation of the ACE system and the release of angiotensin II, which in turn led to vascular endothelial lesions [90]. Additionally, several studies have shown that inhibition of Notch signaling can reduce the activation of M1 macrophages to suppress the inflammation of sepsis [91, 92]. Reports during the COVID-19 pandemic indicate that anti-TNF-*α* drugs increase MAP activity in infected macrophages and the risk of infection by inducing Notch-1 signaling and its downstream effects on IL-6 and MCL-1 [93]. In addition, regulatory dendritic cells (DCregs), the most important specialized antigen-presenting cells, play an important role in the regulation of the pulmonary immune response network, and it was found that activating Notch signaling can induce DC production to attenuate LPS-induced ALI in MSCs therapy for ARDS [94–96]. Moreover, Notch signaling is well studied for lung development and regeneration [97–99]. Different ligands of the Notch pathway may have different effects on type II alveolar epithelial cell (AECII) transdifferentiation: Dlk1 ligands promote the proliferation of AECIIs and inhibit cell transdifferentiation, whereas Jagged1 ligands inhibit the proliferation of AECIIs and promote transdifferentiation to type I alveolar epithelial cells (AECIs). Since AECIIs and AECIs are associated with lung damage and repair in ALI/ARDS, they can provide insight into lung regeneration in late ALI/ARDS studies [100]. As a result, the Notch signaling pathway has two roles in sepsis-induced ARDS. It may promote the inflammatory response through macrophages and inhibit the inflammatory response through DCreg activation. In addition, the Notch signaling pathway is involved in pulmonary development, which may provide ideas for treating ALI/ARDS (Figure 2).

## 3. Conclusions and Perspectives

In summary, multiple signaling pathways are continuously activated in sepsis-induced ALI/ARDS. This disease is mainly regulated through the inflammatory response (inflammatory cell proliferation, apoptosis, and cell polarization) and lung regeneration (AT2 cell proliferation and differentiation). Inhibiting the activation of inflammatory pathways, such as NF-*κ*B signal transduction, and promoting the transduction of anti-inflammatory pathways, such as M2 macrophages, Treg cells, and alveolar regeneration pathways, can reduce the secretion of inflammatory factors and lung edema and promote lung regeneration to cure this disease (Figure 2). Sepsis-induced ALI/ARDS is a critical clinical illness with a highly complex pathogenesis involving multiple cytokines and signaling pathways. Due to its heterogeneity among patients, there is still no effective and specific treatment for the disease. Further research will be necessary to study specific and effective target inhibitors, which depends on the study of genes and proteins involved in the related pathways. However, the study of genes for the diagnosis of inflammatory diseases and proteomics is still in the preliminary stage. In the future, we will be able to analyze the interactions between different proteins and genes through signaling pathways to deepen our understanding of the mechanisms involved. As recent studies have shown, understanding the regulation mechanisms of multiple cellular interactions of this disease is important for the diagnosis and treatment, which can provide a theoretical basis and inspiration for the development of drugs to treat sepsis-induced ALI/ARDS. In addition, the latest experimental research of the sepsis-induced ALI/ARDS model and clinical drug research related to the signaling pathways in ALI/ARDS patients may contribute to a better understanding of the potential of cell therapy related to signaling pathway for COVID-19.

## Data Availability

The data presented in the study may be made available from the corresponding author upon reasonable request.
